# Curse or Blessing? Obesity and Income-Related Inequality in the Chinese Labor Force

**DOI:** 10.3389/fpubh.2021.606634

**Published:** 2021-02-26

**Authors:** Chengxiang Tang, Xiaocong Yang, Fei Peng, Xianglian Hu

**Affiliations:** ^1^Department of Government Administration, School of Public Administration, Guangzhou University, Guangzhou, China; ^2^Department of Economics, School of International Economics and Trade, Shanghai Lixin University of Accounting and Finance, Shanghai, China; ^3^Center for Chinese Public Administration Research, School of Government, Sun Yat-sen University, Guangzhou, China

**Keywords:** obesity, income-related, inequality, Chinese, labor force

## Abstract

China owns a huge labor force of around half billion workers in 2018. However, little is known about the prevalence of obesity and the association between obesity and economic status in this special population. By employing the concentration index (CI) and decomposition analysis, this paper addresses this knowledge gap by using the most recent nationally representative dataset. In specific, this study examines the prevalence of obesity and the socioeconomic gradient in the probability of obesity among Chinese workers between 16 and 65. Our results show that the prevalence of obesity is completely different by using a different measure: the overall prevalence of being general obesity (measured by body mass index, BMI ≥ 28) varies by gender and residency from a minimum of 5.88% to a maximum of 9.46%, whereas abdominal obesity (measured by waist circumference, WCmale ≥ 85 cm & WCfemale ≥ 80 cm) prevalence presents a socking level from 64.53% to 67.69%. Moreover, the results show a pro-rich distribution of obesity (general and abdominal) among male workers (CI_BMI_ = 0.112; CI_WC_ = 0.057) and a pro-poor distribution among female workers (CI_BMI_ = −0.141; CI_WC_ = −0.166). We also find that the direction of the contribution of socioeconomic factors to income-related inequalities in obesity differs by gender. These results have substantial implications for the measurement of socioeconomic inequality in adiposity and for improving health-related policies targeting the Chinese labor force.

## Introduction

Obesity is the most common consequence of overnutrition and has long-term negative health consequences. An increased prevalence of obesity is linked to health risks for numerous non-communicable diseases (NCDs), including cardiovascular diseases, diabetes, and some cancers (1–4). For instance, increased body mass index (BMI) is associated with an increased risk of malignancies caused by alterations in metabolism, insulin levels, and insulin-like growth factors (5). In many developed countries, obesity is a major public health concern and increases the public health burden of NCDs as well as economic costs. According to estimates, the annual medical economic costs of obesity in the United States were over US$140 billion in 2008, and if the prevalence of obesity can be held at 2010 levels, almost US$550 billion in medical costs would be saved by 2030 (4, 6, 7).

The Chinese population has a long history of undernutrition resulting in poor physical and mental health development as well as behavioral abnormalities. Over the last three decades, the Chinese economy has experienced a long period of growth accompanied by a profound transition in the population's lifestyles, wealth, and dietary habits. A wider variety of foods and drinks have become available in the Chinese market, thus increasing rates of consumption of fat-rich and energy-dense foods and sweetened carbonated beverages (8). Over the same period, rapid urbanization and motorization have contributed to decreased rates of physical activity during work and leisure activities (9, 10). Together, these factors have led to a widespread imbalance between energy intake and energy expenditure that has changed the Chinese body weight and composition of nutrition. Consequently, being overweight and obese is becoming increasingly common in both rural and urban areas. The most famous longitudinal study on the Chinese population, the China Health and Nutrition Survey (CHNS), demonstrated that the population's mean BMI has increased over recent decades (11). Specifically, the rates of being overweight and of obesity in Chinese adults were 30.6 and 12.0%, respectively, in 2010 (12).

However, a knowledge gap still exists in the related literature as limited information is available concerning overweight and obese workers in China. Our study contributes to the existing literature in two dimensions. The first contribution of our study is that we applied the most recently available dataset to examine the prevalence of obesity among Chinese workers. To the best of our knowledge, it is the first time to explore the prevalence of both general obesity and abdominal obesity in Chinese workers by using BMI and waist circumference (WC) measures. The majority of studies on obesity have focused on children and the general adult population (4, 13–18). Limited empirical evidence exists on obesity among the labor force, especially in China, which has the largest labor market in the world (19). The existing literature has documented that obesity negatively affects the labor market through two mechanisms. First, the poor health conditions caused by being overweight and by obesity increased rates of absenteeism and presenteeism and, thus, lower the productivity of the labor force (20, 21). Second, workers with higher BMI or perceived overweight-related problems may also be discriminated against by employers, consequently leading to the misallocation of human resources and decreases in workplace productivity (22, 23).

Second, this study provides a useful snapshot of the determinants of socioeconomic gradients in overweight and obesity inequalities. In addition to the prevalence of obesity, we also analyzed socioeconomic gradients by decomposing inequalities in obesity. The factors and processes that affect obesity are multiple and complex, so researchers and policymakers must often understand both the level of the problem and its determinants. A major concern in developed countries is related to the formulation of targeted and effective health policies that mitigate the disproportionate effects of adiposity on people with low incomes or socioeconomic status (SES). The rapid growth of the economy in China has influenced dietary habits, lifestyles, and other health behaviors across different socioeconomic classes.

In China, a few studies have investigated these gradients and the determinants of inequality in BMI-related variables. Using a biased sample, Tafreschi reported that the income-bodyweight gradient changes that have occurred in China from 1991 to 2009 are in line with the reversal hypothesis, which states that in poor or developing societies, obese people tend to be relatively wealthy, whereas in more developed societies, the obese are relatively poor (24). In contrast, our study focuses on a specific population of the workforce using a representative sample, which is an incremental contribution. Although previous study results may only reflect a conditional relationship rather than a causal one, they are meaningful concerning the evolution of socioeconomic health inequalities in China. This is because the shift in higher obesity rates to lower-income individuals may increase the health burden on poor people, thus worsening health inequalities. Another study, using the same dataset, demonstrated that SES is positively related to male BMI and concluded that the relationship between SES and obesity is complicated in China (25). Thus, this paper fills the gap existing in the literature regarding Chinese workers' rates of being overweight, especially in association with their SES.

The paper proceeds as follows: the next section introduces our data, materials, and methods to provide an overview of the employed dataset and a discussion of variables and methodology used in this study. Section three sets out the key results and findings and the final section presents a discussion, outlines the policy implications of the results, and offers concluding points.

## Methods

### Data

This study employed the third wave (2016) of the China Labor-force Dynamics Survey (CLDS), a nationally representative dataset launched by Sun Yat-Sen University in 2012, which was the first national longitudinal survey targeted at the Chinese labor force. It offers longitudinal social survey data with rotating panel design, is conducted every 2 years, and has to date accumulated three waves of data. This dataset implements a probability-proportional-to-size sampling strategy. The population size, administrative units, and SES, which was provided by the local Bureau of Statistics, were used as the main stratification variables. The first wave of the CLDS covered 29 provinces/cities/autonomous regions (excluding Hong Kong, Macao, Taiwan, Tibet Autonomous Region, and Hainan Province) with a total number of 16,253 observations. The next two follow-up waves surveyed the same respondents, plus additional respondents, to provide 23,594 and 21,086 observations, respectively. The dataset of the third wave provides both demographic and socioeconomic information, including measures of employment status, income, household assets, and health outcomes. Of particular relevance for this research is that the CLDS not only uses objective health measurements to generate the key independent variables (e.g., BMI and WC) but also uses the employment status as well as the individual after-tax wage from all sources.

A detailed description of this survey can be found in another study (26). So far, two studies have used this data to examine income-related inequality in health variables. One focused on health care utilization and the other investigated the self-rated health of migrant workers (27, 28). Our study used the most recent (the third) wave of the 2016 CLDS with respondents aged between 16 and 65 years who were working or seeking a job from 7,212 households within 400 communities or villages across the nation, providing a total number of 10,267 valid observations suitable for use. Thus, respondents in this study were part of the current labor force, were aged 16–65 years, and comprised 5,288 males and 4,979 females.

### Dependent and Independent Variables

The first dependent variable in this study was BMI, a widely used general obesity indicator, which is calculated as weight in kilograms divided by the square of height in meters. Another obesity indicator is WC measuring individual waist circumference, which was used in many previous studies as abdominal obesity (20, 29, 30). According to the recommended criteria for Asian and Chinese people (14, 20, 29–31), a BMI of ≥ 28 kg/m^2^ indicates general obesity for both sexes. Regard to the abdominal obesity, we defined respondents' WC of ≥ 85 cm, and WC ≥ 80 cm was considered as abdominal obesity for males and females, respectively. We recoded the continuous indicator BMI and WC into two respective binary health outcomes with a value of 1 indicating being obese (BMI ≥ 28 kg/m^2^; WC of ≥ 85 cm for male and WC ≥ 80 cm for female) and a value of 0 indicating otherwise (BMI < 28 kg/m^2;^ WC of < 85 cm for male and WC < 80 cm for female).

Independent variables comprised of individual after-tax wage, age, gender, health indicators, educational level, marital status, migrant status, region, insurance status, work status, and lifestyle. The key independent variable in this study was the individual after-tax wage, which was used as a measure of individual economic status to analyze the inequality. Its natural logarithm value was employed in the empirical model to examine its effect on BMI and WC. This study also used a binary health outcome of self-rated health (0 = poor; 1 = good) to control the unobserved confounding effects on one's BMI and WC. Education level was coded as primary or below, junior secondary, senior secondary or vocational, and junior college and above. Marital status was classified as single/divorced/widowed, and currently married or cohabits. Previous studies have indicated that the living environment, working situation, and personal lifestyle may affect one's BMI and WC. Thus, we controlled for the migrant effect with four dichotomous variables comprising whether the respondents' migrant statuses were migrant (0 = N; 1 = Y), non-agricultural Hukou (0 = N; 1 = Y), or urban residence (0 = rural; 1 = urban) and if the respondents were located in the south part of China (0 = north; 1 = south). Three types of binary insurance status variables were controlled for medical, retirement, and other types of insurance. Work status was divided into five groups: seeking or willing to seek a job (current not working), employee, employer, self-employed, and agricultural work. The last set of control variables-lifestyle, comprised three binary variables of whether respondents were currently smoking, drinking and participating in physical activities in the past month, with a value of 1 indicating “Yes” and 0 indicating “otherwise.”

### Inequality Measurement

We constructed a concentration curve to illustrate income inequality in the probability of being obese and to calculate the concentration index (CI) to calibrate the degree and significance of income-related inequality regarding the probability of obesity. A concentration curve lying below the line of absolute equality (the 45-degree line) indicated that obesity was concentrated among richer workers, whereas a concentration curve that lies above the line indicates that obesity was concentrated among poorer workers (32). To estimate the degree and significance of inequality, we used a CI that denoted differences in obesity according to individual economic status (32). However, normalization is required because the dependent variables in this study are a binary outcome (e.g., whether the respondent was obese or not), so that the concentration index is quantified in the range −1 to +1. We employ the *Wagstaff-normalization-CI* not only because it has a greater emphasis on relative inequality, but also as it tends to work better for the low-frequency binary outcome (33). The Wagstaff-normalization-CI can be demonstrated by the following:

(1) $$\begin{array}{l} CI_{n}=Cov_{w}\left[\left(\frac{2}{N\mu }\sum_{i=1}^{N}H_{i}R_{i}-1\right)÷\left(1-\mu \right)\right] \end{array}$$

where *CI*_*n*_ denotes the *Wagstaff-normalization-CI* for the probability of being obese (BMI ≥ 28 kg/m^2^ for both sexes, or WC of ≥ 85 cm for male and WC ≥ 80 cm for female) ranging between −1 (perfect pro-poor inequality) and 1 (perfect pro-rich inequality). The *CI* was calculated using the covariance between the probability of being obese and the fractional rank of the individual's after-tax wage. Variable *H*_*i*_ is a binary variable indicating whether the *i*^th^ individual was obese or not, μ stands for the mean rate of actually being obese for the sample, *R*_*i*_ is the fractional rank of the *i*^th^ individual according to his or her after-tax wage, for example, *i* = 1 for the respondent at the bottom of the income distribution (the poorest) and *i* = *N* for those at the top (the richest). *Cov*_*w*_ is the covariance with sampling probability weights, which was provided by the CLDS (34, 35). The 95% confidence intervals for the CIs and associated *p-*values were obtained using the delta method (34, 36, 37). Therefore, a CI significantly smaller than 0 indicated that the greatest proportion of obesity exists among the poorest workers (or we can say poorer individuals were more likely to be obese), namely ‘pro-poor' obesity inequality, whereas a CI significantly >0 indicated that the greatest proportion of obesity exists among the richest workers (or obesity rates occurred more within wealthier individuals), namely ‘pro-rich' obesity inequality (34, 35, 38). We also plotted concentration curves and calculated CIs according to gender subsamples to investigate any related gender differences.

### Decomposition of Inequality in Obesity

In the final stage of the analysis, we conducted a decomposition analysis, following previous studies, to assess the extent to which factors contribute to inequality in the probability of obesity (32, 38–40). Decomposing obesity inequality into the contributions of various explanatory factors was straightforward. According to previously published studies, one's obesity status is not only influenced by demographic factors but also by one's social and cultural environment as well as individual economic status. These factors were classified into 10 main groups: 1) demographic factors (age, gender and self-rated health, etc.); 2) educational level (primary or below, junior secondary, senior secondary or vocational, and junior college and above); 3) marital status (single/ divorced/widowed and currently married or cohabiting); 4) immigrant status (migrant and resident register: *Hukou* status); 5) residency status (rural or urban); 6) regional status (southern or northern China); 7) insurance status (presence of medical insurance, retirement and other insurance); 8) work status (current not working but seeking a job, employee, employer, self-employed, or agricultural worker); 9) lifestyle (whether smoking, drinking, and participated in physical activities in the past month); and 10) individual after-tax wage in the past year. Because the dependent variable was a binary factor with a value between 0 and 1 (BMI and WC), we employed non-linear approximation rather than a linear method (41). Two advantages exist to using this method: (1) the approximation error of a nonlinear model tends to be smaller and (2) compared with linear decomposition, non-linear decomposition more accurately represents partial contributions. The non-linear approximation of a probit model with partial effects evaluated at means can be expressed as follows:

(2) $$\begin{array}{l} H_{BMI\;or\;WC}=\alpha^{m}+\sum_{j}\beta_{j}^{m}X_{ij}\pm \sum_{k}\gamma_{k}^{m}Z_{ik}+\delta^{m}y_{i}+\varepsilon_{i} \end{array}$$

where *H*_*BMIorWC*_ is the obesity indicator(s) defined above; *a*^*m*^ is the intercept; *X*_*ij*_ and *Z*_*ik*_ refer to the *j*^th^ demographic factors (age, gender, and another health indicator, etc.) and *k*^th^ socioeconomic factors (education, marital status, migrant status, residency, region, insurance status, work status, and lifestyle) of the *i*^th^ individual, respectively; *y*_*i*_ denotes individual economic status (measured as the logarithm of individual after-tax wage in the past year); and ε_*i*_ is the error term including approximation errors (35). Additionally, $\beta_{j}^{m}$, $\gamma_{k}^{m}$, and δ^*m*^ are the marginal effects for the aforementioned factors, *dh/dx*_*j*_, *dh/dz*_*k*_, and *dh/dy* of each demographic (*x*), socioeconomics (*z*), and individual economic factor (*y*), respectively, evaluated at sample means. Given Eqs. (1) and (2), the CI can be expressed as follows:

(3) $$\begin{array}{l} CI_{BMI\;or\;WC}=\left(\frac{\delta^{m}\bar{y}}{\mu }\right)C_{y}+\underset{j}{\sum }\left(\frac{\beta_{j}^{m}\bar{X_{j}}}{\mu }\right)C_{j}+\\ \;\underset{k}{\sum }\left(\frac{\gamma_{k}^{m}\bar{Z_{k}}}{\mu }\right)C_{k}+\frac{GC_{\varepsilon }}{\mu } \end{array}$$

where μ is the mean of *H*_*BMIorWC*_. By employing Eq. (1) and adjusting it according to Wagstaff's method (33), *C*_*y*_, *C*_*j*_, and *C*_*k*_ denote the CI of *y*_*i*_, *x*_*j*_, and *z*_*k*_, respectively. *GC*_ε_ represents the generalized CI of the error term ε. Additionally, *y* is the mean individual after-tax wage and $\bar{X_{j}}$ and $\bar{Z_{k}}$ represent the means of the demographic and socioeconomic factors, respectively. Moreover, the products $\left(\beta_{j}^{m}\bar{X_{j}}/\mu \right)C_{j}$, $\left(\gamma_{k}^{m}\bar{Z_{k}}/\mu \right)C_{k}$, and $\left(\delta^{m}\bar{y}/\mu \right)C_{y}$ are the contribution of demographic factor *j*, socioeconomics *k*, and individual wage *y* to the actual concentration index (CI), respectively. A CI was estimated for each of the factors, along with important and percentage contributions to the inequality in the probability of being obese (*CI*_*BMIorWC*_). A positive (negative) contribution indicated that the given factor operated toward a pro-rich (pro-poor) distribution of being obese. We decomposed the CI for the probability of being obese according to the probit model with sample weight applied. Each CI was decomposed into the partial contributions of demographic, educational, marital, migrant, residency, insurance, work, lifestyle, and individual wage factors. Statistical software Stata version 15.1 was used for the analysis.

## Results

### Descriptive Results

Table 1 summarizes the characteristics of the individuals surveyed by the CLDS 2016. There were 10,267 valid respondents for this study. Overall, ~7.3% of the total labor force in China was diagnosed as general obesity, while 44.2% of the sample was diagnosed as abdominal obesity. Specifically, we find that female workers had significantly (*p* < 0.001) lower rates of general obesity (6.4%) than male workers (8.2%). Although the result shows that female workers had slightly higher rates (44.9%) of abdominal obesity than male workers (43.4%), it is statistical insignificance (*p* = 0.237). The above results imply that the measures used for obesity analysis matter and it could result in a completely different conclusion (20, 29, 30). Moreover, we find a piece of preliminary evidence that there is a huge gender wage gap as the mean of individual after-tax wage reveals that male workers have a significantly higher income (26,124.98 Yuan) than female workers (17,190.34 Yuan), and *t*-test results showed that this difference is statistical significance. We observe a similar pattern for most of the other variables except the rate of public social welfare status (i.e., medical and retirement insurance status), as listed in Table 1, indicating a significant difference among the majority factors between female and male workers in China.

**Table 1:** Variable statistics description, full, female, and male samples^a^.

**Variables^**b**^**	**Female**	**Male**	**Total**	
	**(N = 4,979)**	**(N = 5,288)**	**(N = 10,267)**	***p-*value^**c**^**
**Key variables**				
General obesity (BMI≥28)	319 (6.4%)	430 (8.1%)	749 (7.3%)	0.001
Abdominal obesity4 (WCF≥80; WCM≥85)	1,334 (44.0%)	1,350 (43.6%)	2,684 (43.8%)	0.757
**Individual after-tax wage**				<0.001
Mean (SD)	17,190.34 (32,582.33)	26,124.98 (40,315.03)	21,792.11 (37,037.12)	
Min, Max	0.0, 800000.0	0.0, 960000.0	0.0, 960000.0	
**Demographic factors**				
Age				<0.001
Mean (SD)	43.27 (11.54)	44.38 (11.94)	43.84 (11.76)	
Min, Max	16.0, 65.0	16.0, 65.0	16.0, 65.0	
Gender (0 = F; 1 = M)				<0.001
Female	4,979 (100.0%)	0 (0.0%)	4,979 (48.5%)	
Male	0 (0.0%)	5,288 (100.0%)	5,288 (51.5%)	
Self-rate health (0 = Poor; 1 = Fair)	4,277 (85.9%)	4,730 (89.4%)	9,007 (87.7%)	<0.001
**Socioeconomic factors**				
Educational status				<0.001
Primary or below	2,035 (40.9%)	1,313 (24.8%)	3,348 (32.6%)	
Junior secondary	1,551 (31.2%)	2,161 (40.9%)	3,712 (36.2%)	
Senior secondary or vocational	627 (12.6%)	1,029 (19.5%)	1,656 (16.1%)	
Junior college and above	766 (15.4%)	785 (14.8%)	1,551 (15.1%)	
Married (0 = N; 1 = Y)	4,466 (89.7%)	4,504 (85.2%)	8,970 (87.4%)	<0.001
Migrant (0 = N; 1 = Y)	1,833 (36.8%)	753 (14.2%)	2,586 (25.2%)	<0.001
Hukou (0 = Agr;1 = Non-agr)	1,198 (24.1%)	1,427 (27.0%)	2,625 (25.6%)	0.001
Live in urban (0 = N; 1 = Y)	1,709 (34.3%)	1,947 (36.8%)	3,656 (35.6%)	0.008
South China	2,940 (59.0%)	3,173 (60.0%)	6,113 (59.5%)	0.324
Medical insurance (0 = N; 1 = Y)	4,564 (91.7%)	4,863 (92.0%)	9,427 (91.8%)	0.582
Retirement insurance (0 = N; 1 =Y)	3,303 (66.3%)	3,527 (66.7%)	6,830 (66.5%)	0.700
Other insurances (0 = N; 1 = Y)	974 (19.6%)	1,148 (21.7%)	2,122 (20.7%)	0.007
Working status				<0.001
Not working	499 (10.0%)	334 (6.3%)	833 (8.1%)	
Employee	1,857 (37.3%)	2,176 (41.1%)	4,033 (39.3%)	
Employer	72 (1.4%)	155 (2.9%)	227 (2.2%)	
Self-employ	437 (8.8%)	719 (13.6%)	1,156 (11.3%)	
Agriculture work	2,114 (42.5%)	1,904 (36.0%)	4,018 (39.1%)	
**Lifestyle (N** **=** **0; Y** **=** **1)**				
Smoking	77 (1.5%)	2,789 (52.7%)	2,866 (27.9%)	<0.001
Drinking	171 (3.4%)	1,936 (36.6%)	2,107 (20.5%)	<0.001
Physical activities	1,357 (27.3%)	1,537 (29.1%)	2,894 (28.2%)	0.041

*Source: CLDS 2016*.

a Respondents are laborforces aged 16–65 in China and results are adjusted by sampling weights;

b for continuous variables the mean and standard deviation (in parentheses) are presented, while for categorical variables the number of respondents and percentage of the sample (in parentheses) are presented;

c *for continuous variable the ANOVA has been used while for categorical variables the chi-square test has been used to show the between-groups-difference; 4) due to data availability, there are total 6,132 respondents reported their waist circumference (WC)*.

### Prevalence of Obesity Among Workers by Gender

In Table 2, the age-adjusted prevalence of being overweight and of obesity, as well as their 95% confidence intervals, are presented as totals and by gender for both rural and urban areas. Both BMI and WC measures are listed. All estimates were calculated using a weighting variable to accurately represent the working population of China; thus, the estimates were made to represent China's 454.2 million workers, comprising 218.2 million females and 236.0 million males. Table 2 shows that the prevalence of being general overweight (BMI ≥ 28 kg/m^2^) among the labor force was 21.98% (rural) and 24.8% (urban), whereas the prevalence of general obesity among workers was 6.265% (rural) and 8.153% (urban). Both results imply that urban workers were more likely to be obese than rural workers. In terms of the results of overall rates of abdominal overweight among Chinese workers were 19.38% (rural) and 17.19% (urban), while 64.53% (rural) and 67.69% (urban) were diagnosed as abdominal obesity. The above results not only reconfirmed that different obesity indicators produce completely different conclusions, but also pointed out that one-fifth of the labor force in China is overweight, which may be related to the rapid development of China's economic and living standard as well as the increased total caloric intake in the past decades (24).

**Table 2:** Prevalence of being overweight and of obesity among workers by residency.

	**Pre-obesity or overweight**			**Obesity**		
	**(age-adjusted estimate) % (95% CI)**			**(age-adjusted estimate) % (95% CI)**		
	**Both sexes**	**Male**	**Female**	**Both sexes**	**Male**	**Female**
**BMI**^**a**^						
Rural	21.86	23.79	20.13	6.226	6.589	5.89
	[20.36,23.36]	[21.64,25.94]	[18.23,22.03]	[5.458,6.993]	[5.480,7.698]	[4.877,6.904]
Urban	24.84	28.81	19.79	8.205	9.486	6.306
	[22.49,27.19]	[26.12,31.51]	[16.90,22.68]	[6.579,9.832]	[7.372,11.60]	[4.439,8.173]
*t*-test^c^	−2.092	−2.869	0.189	2.159	−2.382	−0.383
*p-value*	0.037	0.004	0.85	0.031	0.017	0.702
**WC**^**b**^						
Rural	19.31	19.12	19.07	64.75	65.14	64.83
	[17.73,20.89]	[17.14,21.10]	[17.05,21.10]	[62.65,66.84]	[62.47,67.82]	[61.81,67.84]
Urban	17	18.46	16.67	68.31	69.14	67.65
	[15.14,18.86]	[16.11,20.81]	[14.29,19.06]	[65.59,71.04]	[66.21,72.07]	[64.34,70.95]
*t*-test	1.859	0.419	1.499	−2.042	−1.995	−1.238
*p-value*	0.063	0.675	0.134	0.041	0.046	0.216

*Sources: CLDS 2016, respondents are labor forces aged 16–65 in China and results are adjusted by sampling weights*.

a BMI ≥ 24 kg/m^2^ for overweight, BMI ≥ 28 kg/m^2^ for obesity;

b For female, WC ≥ 75 cm for overweight, WC ≥ 80 cm for obesity; for male, WC ≥ 80 cm for overweight, WC ≥ 85 cm for obesity;

c *t-test for the prevalence difference (overweight or obesity) between rural and urban subsamples*.

### Inequality and Decomposition Analysis

**Figure 1:**
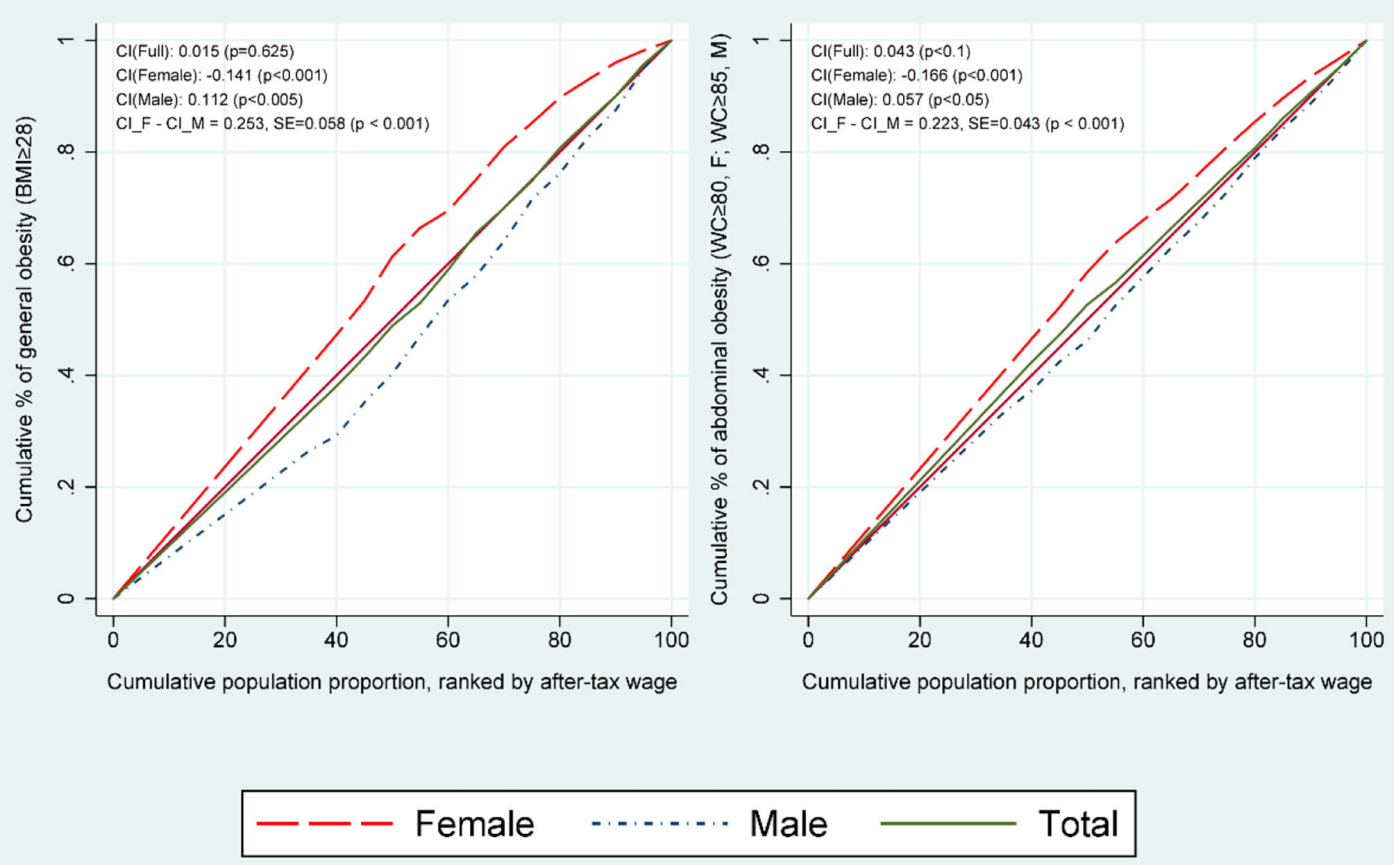
Concentration curve for the probability of being general or abdominal obese (BMI ≥ 28; WC≥80 for females or WC≥85 for males) by total, female, and male works, China, 2016. X-axis *represents the cumulative population proportion, ranked by individual annual after-tax wage; respondents are labor forces aged 16–65 in China and results are adjusted by sampling weights*.

Figure 1 displays the concentration curves for the probability of being general obese (BMI ≥ 28) and abdominal obese (WC ≥ 80, F; WC ≥ 85, M) for the total, male, and female workers. We did not observe any significant pro-rich or pro-poor distribution of general obesity in the overall (total) labor force population when considering the BMI measures. However, a significant pro-rich distribution was observed in male workers (CI = 0.112, *p* < 0.001). By contrast, a pro-poor distribution was observed in female workers (CI = −0.141, *p* < 0.01) with a slightly larger CI value than existed for males (|−0.141| > |0.112|). The aforementioned results showed that the probability of being general obese is biased toward wealthier male workers, whereas poorer female workers are more likely to be general obese. Regarding the WC measures, the result shows that a slightly significant pro-poor distribution of abdominal obesity (CI = −0.043, *p* < 0.1) in the overall labor forces. Specifically, as same as the BMI measures, we also divided into male and female subsamples, and the result shows a significant pro-rich (CI = 0.059, *p* < 0.1) and a pro-poor (CI = −0.166, *p* < 0.001) distribution in male workers and female workers, respectively. In summary, we employed both BMI and WC to measure respondent obesity status and their particular relation with individual after-tax wage, even though the values of CIs are different, the results show that the income-related obesity distribution of male worker presents a pro-rich distribution, while for the female worker a pro-poor distribution is shown. The result suggests that male workers with higher incomes are more likely to become obese, and conversely, female workers with lower incomes are more likely to become obese.

Table 3 lists the detailed contributions of all factors to inequality in the probability of being obese for male and female workers in China. A positive (negative) partial contribution indicates that the factor increases (decreases) the total inequality in income-related obesity (*CI*_*BMI*_ *or CI*_*WC*_), with positive (negative) percentages indicating increases (decreases) in percentages. We observe that individual economic status (logarithmic transferred individual after-tax wage in the past year) played a key role for pro-rich general obesity in males (77.68%) and played a similar role for pro-poor distribution in females (41.56%). However, its percentage contribution decreases significantly in the abdominal obese measures for both male workers (12.13%) and female workers (37.37%). We also find that being educated to the senior secondary or vocational and above contributed to a pro-poor probability of being obese, which was most pronounced for female workers in both generals (15.72 and 36.88%) and abdominal (9.43 and 40.86%) obesity. However, the educational factors were less of a contributing factor and played an opposite direction for male workers (from a minimum |−0.702| to a maximum |−21.06|). Moreover, some contributing factors displayed different roles for female and male workers since their CI curves operate the opposite distribution. For instance, another contributor to increased income-related inequality in general and abdominal obesity was region status for female respondents (south China, 14.99 and 7.510%), whereas this factor was decreased the income-related inequality in both obesity indexes and more influential for male workers (south China, −26.20 and −49.85%), implying that the male labor forces who were living in the south part of China play a significant role in declining the pre-rich income-related obesity distribution. In addition, one's employment status, such as being an employee, was the main contributor for both male and female respondents in general obesity (44.25 and 54.78%) but contributed less to abdominal obesity (16.80 and 7.0%). However, the results show that involvement in agriculture work decreases the income-related (general and abdominal) obesity inequality in both male and female workers, especially for female workers (−40.13 and −31.92%). Similar results with variations (positive or negative contributions) were also observed in the other group of variables, such as other migrant status indicators, insurance status indicators, and lifestyle, etc. The details are listed in Table 3.

**Table 3:** Detailed contributions to inequality in the probability of being obese by demographic and socioeconomic factors, and female and male labor forces.

**Contribution and percentage contribution**	**BMI**				**WC**			
	**Female**		**Male**		**Female**		**Male**	
	**Contr. to CI**	**% contr. to CI**	**Contr. to CI**	**% contr. to CI**	**Contr. to CI**	**% contr. to CI**	**Contr. to CI**	**% contr. to CI**
**CI**	−0.129	100	0.112	100	−0.092	100	0.033	100
Residual	−0.056	43.193	−0.004	−3.147	−0.010	10.640	−0.008	−23.098
After-tax wage (log)	−0.054	41.555	0.087	77.676	−0.034	37.365	0.004	12.130
**Demographic & socioeconomic factors**								
Age	−0.003	2.107	0.018	15.649	−0.037	40.405	−0.003	−9.817
SRH	0.000	−0.381	−0.003	−2.795	−0.003	2.889	−0.004	−10.913
**Education level (ref: primary or below)**								
Junior secondary	0.002	−1.794	0.002	2.044	0.001	−1.097	−0.000	−0.980
Senior secondary or vocational	−0.020	15.722	−0.001	−0.702	−0.009	9.431	−0.002	−6.255
Junior college and above	−0.048	36.878	−0.024	−21.063	−0.038	40.858	−0.005	−14.255
**Marital status (ref: never married/divorced/widowed)**								
Currently married or cohabitating	−0.001	0.966	−0.003	−2.263	−0.000	0.034	−0.001	−2.282
**Migrant status**								
Migrant	0.004	−3.366	−0.005	−4.684	−0.002	1.876	−0.013	−39.840
Non-agricultural Hukou	0.028	−21.323	0.004	3.416	−0.004	4.291	0.014	41.820
Residency	0.006	−4.995	0.004	3.333	0.014	−15.187	0.009	27.701
Region	−0.019	14.994	−0.029	−26.199	−0.007	7.510	−0.016	−49.849
**Insurances**								
Medical insurance	0.000	−0.287	0.001	0.608	0.000	−0.225	0.001	2.002
Retirement insurance	0.002	−1.867	0.005	4.596	0.000	−0.292	0.001	2.833
Other insurances	0.037	−28.675	−0.025	−22.647	0.018	−19.213	0.004	12.074
**Work status (ref: not working)**								
Employee	−0.057	44.254	0.061	54.789	−0.015	16.803	0.002	7.002
Employer	−0.000	0.232	0.002	1.854	−0.000	0.074	0.000	1.201
Self-employ	0.000	−0.164	−0.002	−1.444	0.001	−1.489	0.001	2.322
Agriculture work	0.052	−40.127	−0.011	−9.814	0.029	−31.921	0.037	114.884
**Lifestyle**								
Smoking	−0.000	0.226	0.002	1.849	0.000	−0.330	0.000	0.231
Drinking	−0.000	0.007	0.000	0.145	−0.000	0.011	0.000	1.351
Physical activities	−0.004	2.847	0.032	28.798	0.002	−2.433	0.010	31.736

*Sources: CLDS 2016*.

*Decomposition based on the results of probit model. Sample weights applied. CIs were calculated and adjusted by the method proposed by Wagstaff (33), as same as shown in Figure 1*.

**Figure 2:**
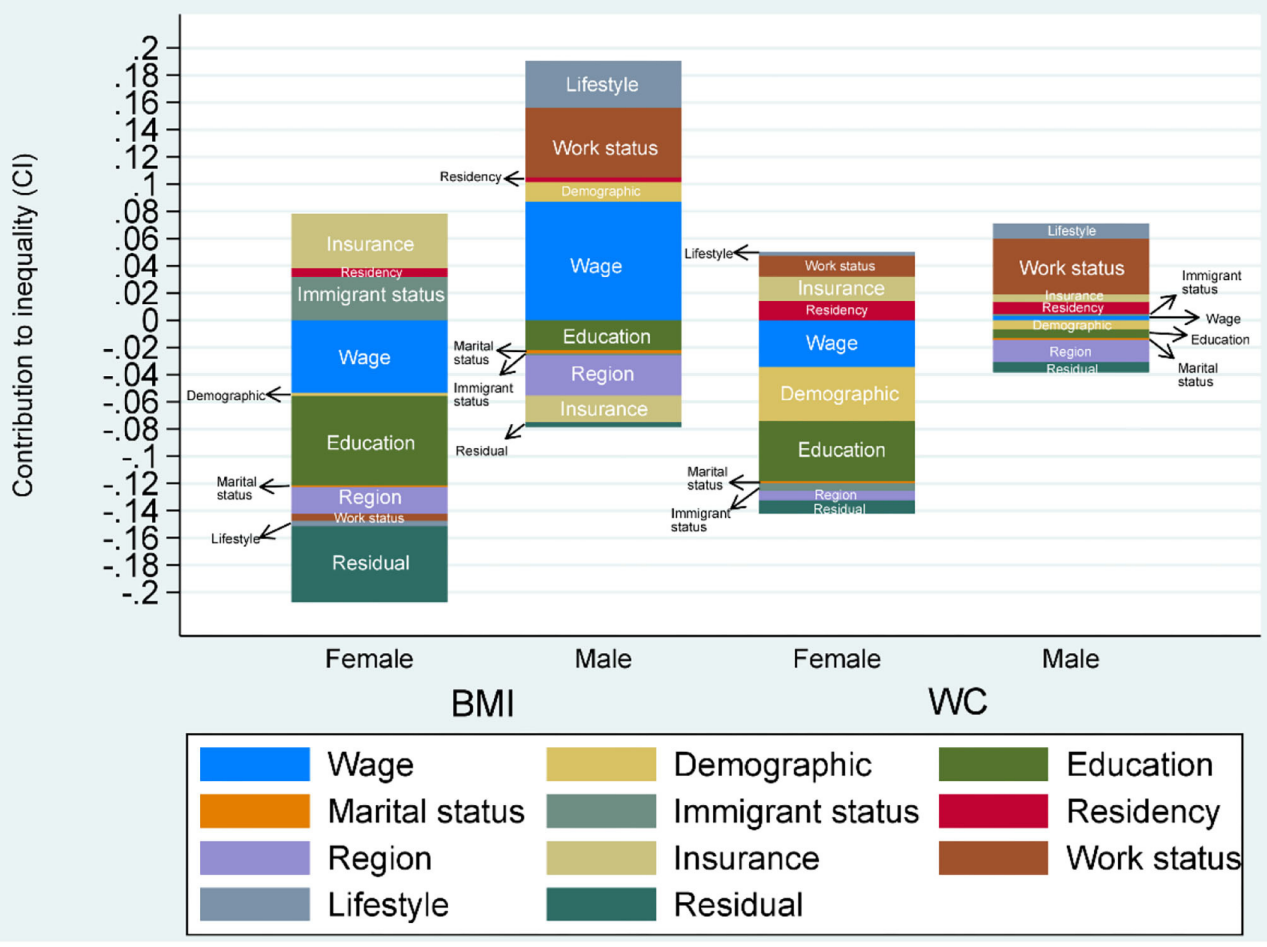
Contributions of Demographic and socioeconomic factors to CI for the probability of obesity (BMI ≥ 28; WC ≥ 80 for females or WC ≥ 85 for males) in China by female and male workers.

Figure 2 is a graphical depiction of inequality in income-related general and abdominal obesity decomposed into 11 determinants. The results are separated by gender as per Table 3, and the determinants comprise demographics (age, gender, and health indicators), educational level, marital status, migrant status, residency status, region, insurance status, working status, lifestyle, individual economic status (wage), and residual terms. Figure 2 indicates that inequality was considerably higher for females than for males, as same as results shown in Figure 1 and Table 3.

Figure 2 suggests that demographic factors contributed the least to the income-related inequality in general and abdominal obesity among female and male workers. In addition, as same as listed in Table 3, this indicates that individual economic status and other socioeconomics factors were the main contributors to the income-related inequality in general and abdominal obesity for both female and male workers. However, some factors were associated with increased inequality in income-related obesity for male workers but with decreased inequality for females. Other socioeconomics contributors, such as insurance, immigrant status, work status, education level, work status, residency status, lifestyle, and region were also drivers of this difference. Specifically, for the female subsample, contributors such as immigrant, insurance and residency statuses accounted for reducing the pro-poor concentration of general obesity, whereas the other contributors, especial educational level, work status, wage, and unexplainable factors (residuals) operated to increase the pro-poor concentration in general obesity. For males, the distribution of pro-rich general obesity was driven by factors of residency, work status, lifestyle, and individual economic status. However, the contribution of education, immigrant, region, insurance, and marital status factors reduced the pro-rich inequality in general obesity. We can find a similar result but with some differences in abdominal obesity among male and female subsample, respectively.

## Discussion

This paper reveals the fact that the overall prevalence of being overweight and obesity in China is at a high-level as a developing country. The overall prevalence of being general obesity (measured by BMI) varies by gender and residency from a minimum of 5.88% to a maximum of 9.46%. In contrast, abdominal obesity (measured by WC) prevalence presents a socking level from 64.53 to 67.69%. Our study is consistent with another study that also revealed the prevalence of overweight and obesity varies greatly among different population subgroups (42). We find that there is significant income-related inequality in general and abdominal obesity thresholds for both female and male workers in China. The lower-income female workers are more likely to experience obesity than the richer female workers, whereas wealthier male workers are more likely to be obese than poorer male workers. We also noticed that the different effects for male and female workers counteract each other, thus, the overall result of the Chinese labor force does not show any pro-rich or pro-poor concentrations of income-related inequality regarding the probability of general obesity, but our result presents that there are slightly significant pro-poor concentrations of income-related inequality in abdominal obesity among all Chinese labor forces.

Additionally, the decomposition analysis revealed that educational level and individual after-tax wage in the past year were the main contributors to inequality in general and abdominal obesity for females, whereas working status, income, and lifestyle were the main contributors to inequality in general and abdominal obesity for males. Moreover, contributors to income-related obesity inequality, such as immigrant, insurance and residency statuses, were determined to reduce the pro-poor concentration of general obesity in female workers. These two factors also decreased the pro-rich concentration of general obesity in males. The aforementioned results may indicate that, besides income level, other social factors such as educational level, immigrant and insurance statuses contributed to the general and abdominal obesity inequality significantly.

Our study represents one of the first attempts to characterize the association between obesity and socioeconomic parameters in the Chinese working population. Previous studies from other countries, such as the United States, have highlighted the medical costs and loss of productivity caused by increased rates of being overweight and obesity and have estimated that the aggregated annual cost attributable to obesity among full-time employees is US$73.1 billion (6, 43). Numerous studies have shown that obesity or being overweight among workers may cause adverse occupational consequences such as absenteeism and presenteeism, work limitations, and workplace impairment (44–46). Future studies should explore possible reasons that the labor force, which was the target population in our study, is disproportionately affected by central adiposity. Moreover, a large proportion of the working population was observed to be overweight or pre-obese, whereas the prevalence of obesity was considerably lower. This significant distinction between being overweight and being obese may indicate rapid growth in obesity in workers, rather than being an indicator of the validity of the cut-off point concerning defined body fat.

The inequalities in obesity for men were moderately less than those for women. This finding is consistent with a related theory about income and obesity (47). A related study also reported a significant pro-rich inequality for females (48), which is inconsistent with our results, indicating a pro-poor distribution among female workers. Our findings might be attributed to two factors. The first is that rich female workers might engage more in fitness-related activities and the second is that poor female workers might have an excessive intake of total carbohydrates.

Additionally, the decomposition analysis also indicated that individual income and education were the main explanatory factors of both general and abdominal obese inequality, especially for females. The possible explanation for these two significant factors might be contributed by the “one-child” policy, which to improve the educational attainment and earnings of Chinese females in the past decades, or the so-called “missing girls' effect” (49, 50). Moreover, a recent report shows that the percentage of females enrolled in masters-level postgraduate programs increased from 50.36% in 2010 to 53.14% in 2016, while the percentage of females enrolled in undergraduate programs increased from 49.68% in 2010 to 53.44% in 2016 (51). Considering our observed samples are mainly from the latest labor force in 2016, which means that they were born in the 80s and 90s–the “one-child” policy generation. It is also explaining why the educational level at junior college and above among female workers is greater than male workers as Table 1 shows. Therefore, compared with male workers, education plays a more important role in reducing obesity income-related inequality among female workers. Our results are also in line with those of previous studies that have indicated how income and educational attainment strongly related to levels of obesity (52). Other factors, such as working status and lifestyle, were evident contributors to inequality in obesity for male workers. It is possible as different work status and lifestyle has been proved that strongly correlated with one's weight, especial for males (53, 54). In addition, there is a related paper revealed that a majority of the social gradient concerning health inequality could be explained by the work environment and lifestyle factors (55).

This study also has a couple of limitations. First, two binary obesity indicators measured using BMI and WC may not completely present all aspects of human obesity index in the labor force (18). For example, BMI and WC do not estimate lean muscle mass. However, an advantage is that both BMI and WC are highly correlated with body fat percentage and is widely used to define obesity. Second, although the CI has been widely applied in the measurements of inequality (56), intense debate exists concerning the characteristics and value judgments of this index's spectrum (57, 58). Nevertheless, no consensus exists about the indicators that should be used to evaluate inequalities in health or health-related lifestyle (59). Third, our study presented evidence of correlation instead of causality. Finally, our data is limited to the Chinese labor force, which we considered important as it accounts for around one-fifth of the world's labor force.

## Conclusion

To summarize, this study is the first to describe the prevalence of overweight and obesity in the Chinese labor force. Our research is relevant because we focused on the population group that is most crucial to productivity and economic development. The results also have substantial implications for the measurement of socioeconomic inequalities in adiposity. For example, income factors play opposite roles in contributing to inequalities in obesity between female and male labor forces in China. Educational level, however, plays a significant role in decreasing the obesity income-related inequality for both sexes, especially for female workers. The possible reason could be contributed by the “one-child” policy or the so-called “missing girls' effect.” Apart from individual income, education, and lifestyle, we observe that work-related factors, such as work status, contribute to inequality in obesity. For health policymakers, understanding the determinants of being overweight and of obesity may help in designing interventions that promote health and fitness and, consequently, improve associated labor outcomes.

## Data Availability Statement

Publicly available datasets were analyzed in this study. This data can be found at: http://www.cnsda.org/index.php?r=projects/view&id=75023529.

## Ethics Statement

Ethical review and approval was not required for the study on human participants in accordance with the local legislation and institutional requirements. Written informed consent for participation was not required for this study in accordance with the national legislation and the institutional requirements.

## Author Contributions

CT conceived and designed the experiments. XY performed the experiments and analyzed the data. CT and XY wrote the paper. XY and FP restructured, polished, and revised the paper. XH contributed this work by providing data access. All authors read and approved the final manuscript.

## Conflict of Interest

The authors declare that the research was conducted in the absence of any commercial or financial relationships that could be construed as a potential conflict of interest.
